# Supplementary material for: Neuroprotective effects of Sonic hedgehog agonist SAG in a rat model of neonatal stroke
Source: Pediatr Res. 2021 Mar 2;90(6):1161–70. doi: 10.1038/s41390-021-01408-7 (PMC8410885; doi:10.1038/s41390-021-01408-7)
Supplement: Supplementary file 1 — Supplementary Material [file 41390_2021_1408_MOESM1_ESM.pdf]

Supplemental Material

Supplementary Figure S1: Animal usage flow chart

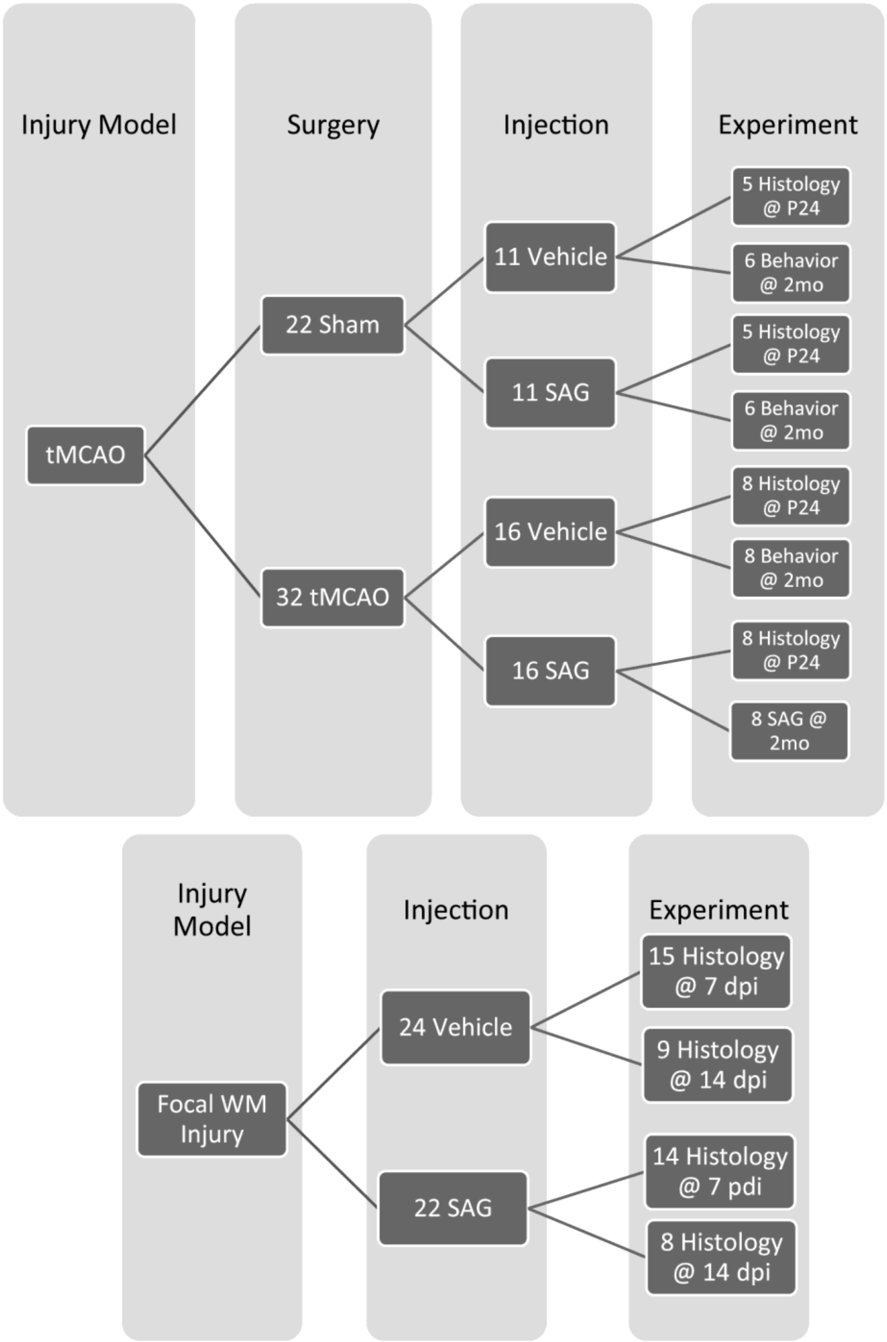

Total number of animals used for tMCAO and focal demyelination experiments, and their allocations.

Supplementary Figure S2: Duration of SAG bioactivity *in vivo*

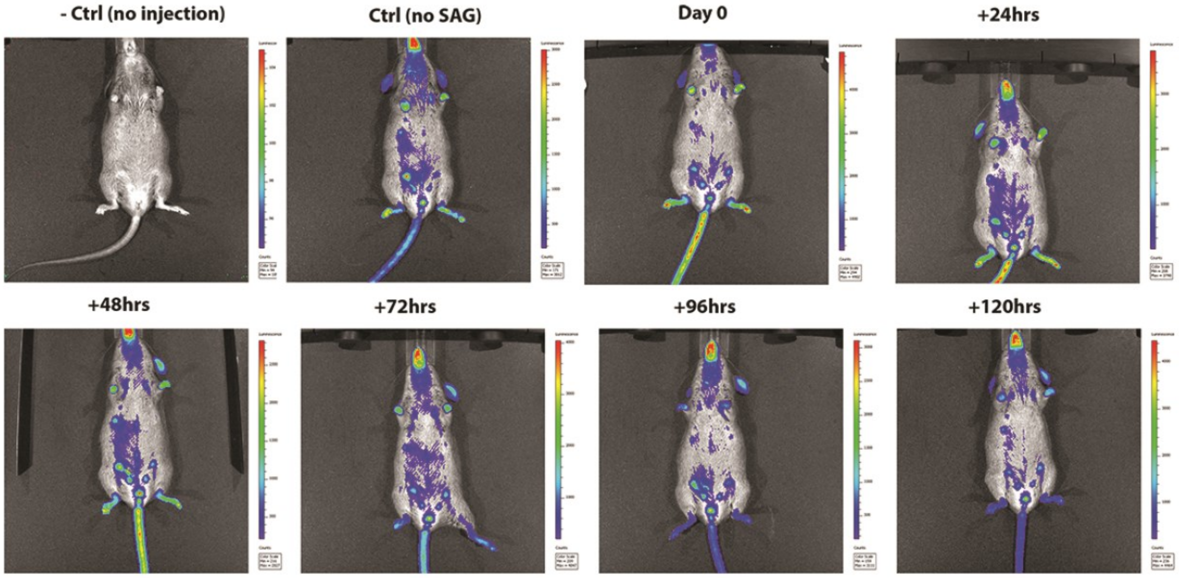

SAG injection (50mg/kg body weight) into *Gli-Luciferase* reporter mice shows increase in Shh downstream target *Gli1* in the limbs and tail lasting between 48 and 72 hrs.
